# Editorial: Epigenetic mechanisms and epigenetic-based therapies in cardiometabolic and vascular disease

**DOI:** 10.3389/fgene.2023.1233096

**Published:** 2023-06-23

**Authors:** Yanshuo Han, Chen Chen, Jian Zhang

**Affiliations:** ^1^ School of Life and Pharmaceutical Sciences, Dalian University of Technology, Panjin, China; ^2^ School of Biomedical Sciences, University of Queensland, Brisbane, QLD, Australia; ^3^Department of Vascular Surgery, The First Hospital of China Medical University, Shenyang, China

**Keywords:** epigenectic regulation, epigenetic therapeutics, abdominal aort aneurysm, cardiometabolic and vascular disease, noncoding RNA

Cardiometabolic and vascular disease (CMVD) and its complications pose significant global health concerns, leading to increased incidence and mortality rates. Epigenetic regulation has emerged as a key player in CMVD, offering new insights into its pathogenesis and potential therapeutic interventions. In recent years, the role of epigenetic regulation in CMVD has gained considerable attention (Xia et al., 2019) (Davis and Gallagher, 2019). This Research Topic explores the intricate connections between epigenetic modifications, CMVD development, and therapeutic strategies. By investigating histone modifications, DNA methylation, non-coding RNAs, and RNA methylation, we aim to identify novel epigenetic markers and develop targeted therapies for managing CMVD.

This Research Topic delves into various aspects of epigenetic regulation in CMVD, including specific modifications and regulators (Wang et al.) involved in its pathogenesis. It also explores the potential of epigenetic enzymes (Han et al., 2016) as therapeutic targets and highlights groundbreaking discoveries in the field. Meanwhile, examining the influence of epigenetic regulation on additional cardiovascular diseases, such as aortic aneurysm (He et al., 2021), aortic dissection (Zhang et al., 2021), and their pathogenic mechanisms. Furthermore, it examines the influence of epigenetic regulation on other cardiovascular diseases and investigates the effects of epigenetic modifications on cell phenotypes. In Xu’s review, they summarized numerous epigenetic modifications have suggested potential roles in cell phenotypes during AAA development, e.g., death and phenotypic switch of vascular smooth muscle cells, infiltration of inflammatory cells, endothelial dysfunction, and perivascular adipose tissue (PVAT) dysfunction (Xu et al.). Another study analyzed the cluster specific cell phenotype markers identified in scRNA-seq dataset; they revealed high proportion of structure cells in abdominal aortic tissues in Takayasu arteritis patients by deconvolution of bulk RNA-seq dataset (Yuqing et al.).

The identification and clinical significance of risk factors associated with diagnosing and predicting CMVD prognosis are explored in this Research Topic. Yuqing et al. suggested interleukins may play a critical role in the pathogenesis of Takayasu arteritis (Yuqing et al.). Additionally, novel insights into histone modification, RNA modifications (Wu et al., 2022), and non-coding RNAs (Xu et al.) are discussed, highlighting their relevance in basic and translational research related to vascular disorders. The dysregulation of epigenetic and transcriptional processes in CMVD is examined, uncovering potential therapeutic opportunities. These dysregulations include m6A modification and its correlation with immune infiltration in abdominal aortic aneurysm (AAA). Individualized treatment possibilities based on m6A modification are also discussed. In Tian’s study, they developed a reliable AAA-related disease model for predicting immunity and m1A, m5C, m6A and m7G epigenetic regulation. Meanwhile, Tian et al. suggested that the pathogenic roles of four model RNA methylation genes, UBE2K, TMEM230, VAMP7, and PUM2, might play an important role in AAA (Tian et al.).

The contributions within this Research Topic highlight the critical role of epigenetic regulation in CMVD pathogenesis and provide avenues for targeted interventions. By deciphering the complex interactions between epigenetic modifications and CMVD, we are paving the way for personalized approaches to diagnosis, risk stratification, and treatment. The knowledge gained from this Research Topic will undoubtedly contribute to improving cardiometabolic health and reducing the burden of CMVD on a global scale.
